# Effects of preoperative systemic administration of tranexamic acid alone on postoperative inflammation and pain in total hip arthroplasty: a retrospective cohort study

**DOI:** 10.1186/s42836-025-00320-3

**Published:** 2025-07-04

**Authors:** Fumihiro Mukasa, Tomonori Baba, Koju Hayashi, Taiji Watari, Muneaki Ishijima

**Affiliations:** Department of Orthopedic Surgery, Juntendo University, 2-1-1 Hongo, Bunkyo-Ku, Tokyo, 113-8431 Japan

**Keywords:** Inflammation, Pain, Total hip arthroplasty, Tranexamic acid

## Abstract

**Background:**

Recent studies have demonstrated that tranexamic acid (TXA) effectively reduces postoperative blood loss after total hip arthroplasty (THA) and is a safe treatment option. However, the anti-inflammatory effect of using TXA without dexamethasone (DEX) in THA remains unclear. In this study, we evaluated the anti-inflammatory effects, postoperative pain reduction, hidden blood loss (HBL), and postoperative complications associated with the use of TXA in THA.

**Methods:**

This retrospective cohort study included 126 patients who underwent primary THA via a direct anterior approach (DAA) between January 1, 2023, and February 29, 2024. Patients were divided into two groups based on the administration of TXA (1000 mg IV preoperatively): Group A (with TXA) and Group B (without TXA). The postoperative inflammatory response (C-reactive protein [CRP] levels) and pain (numerical rating scale [NRS]) were assessed on postoperative days (PODs) 1, 3, and 7. HBL was assessed on PODs 3 and 7. Postoperative complications were counted based on occurrences from the postoperative period until discharge.

**Results:**

CRP levels were significantly lower on POD 1 in Group A than in Group B (*P* = 0.002). Postoperative pain levels in Group A peaked later, with a significant reduction in the NRS score on POD 3, compared with that in Group B (*P* = 0.031). HBL in Group A was significantly reduced on PODs 3 (*P* < 0.001) and 7 (*P* = 0.013) compared to that in Group B. Postoperative complications did not differ significantly between Groups A and B.

**Conclusion:**

TXA can effectively reduce postoperative blood loss, inflammation, and pain in patients undergoing THA without postoperative complications. Using TXA alone remains a highly effective and practical approach for improving early postoperative outcomes in patients undergoing THA.

## Background

Hip osteoarthritis frequently results in chronic pain, restricted joint mobility, muscle weakness, and a decline in overall quality of life (QOL) [1]. In advanced cases, total hip arthroplasty (THA) is often performed to alleviate pain, restore function, and improve QOL. However, perioperative bleeding and inflammation can delay rehabilitation and affect recovery outcomes [2].

Various minimally invasive techniques have been employed to treat perioperative bleeding, including the muscle-sparing approach, autologous blood transfusion, and pharmacological treatments [3, 4]. Tranexamic acid (TXA) effectively reduces postoperative blood loss after THA and is safe for use [5–8]. Additionally, TXA has shown anti-inflammatory effects, likely through the inhibition of plasmin activation, thus reducing complement and immune cell activation during cardiovascular surgery [9, 10].

In THA, the anti-inflammatory effects of the combined use of TXA and dexamethasone (DEX) have been reported [11]; however, no studies have compared the effects of TXA only with those observed in the absence of TXA use in the context of THA using the direct anterior approach (DAA-THA). Therefore, to our knowledge, this study is the first to investigate the standalone anti-inflammatory role of TXA in DAA-THA.

Based on these findings, we hypothesized that TXA alone could not only reduce blood loss in DAA-THA but also suppress postoperative inflammation and pain. Thus, this study aimed to evaluate the anti-inflammatory and pain-reducing effects of TXA after DAA-THA.

## Methods

This was a single-center retrospective cohort analysis of primary THA. This study was approved by the relevant institutional review board (E24-0044). Instead of obtaining patients’ informed consent for data use directly, an opt-out system on the institutional website was used.

### Participants

**Fig. 1:**
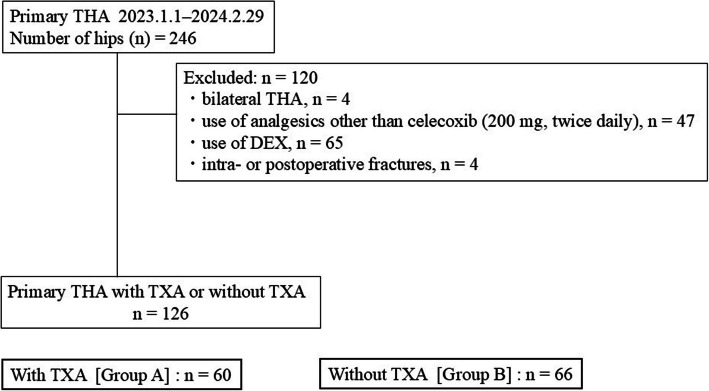
Flowchart of the inclusion and exclusion of patients in this study. THA: total hip arthroplasty, TXA: tranexamic acid, DEX: dexamethasone

The medical records of 264 patients who underwent THA for osteoarthritis between January 1, 2023, and February 29, 2024, were retrospectively reviewed. Exclusion criteria were: (1) bilateral THA, (2) use of analgesics other than celecoxib (200 mg, twice daily; Pfizer, New York, NY, USA) (e.g., continued postoperative use of preoperatively prescribed medications such as mirogabalin for lumbar spinal stenosis) (3) use of DEX, (4) intraoperative or postoperative fractures. The final cohort of 126 primary THA cases was analyzed using the DAA. Figure 1 shows the study flowchart.

### Medication administration, surgical procedure, and implant information

TXA was not administered to patients with THA at our institution between January 1 and June 30, 2023. However, from July 1, 2023, to February 29, 2024, TXA was routinely administered intravenously as a single dose of 1000 mg immediately before surgery. All surgeries were performed under general anesthesia, and the patients received postoperative intravenous patient-controlled analgesia. DEX was administered at 6.6 mg intraoperatively at the discretion of the anesthesiologist to prevent postoperative nausea [12].

All procedures used the direct DAA via the distal extension of the Smith–Petersen approach [13]. Surgeries were performed by five experienced orthopedic hip specialists or supervised residents with experience in more than 40 cases of the DAA. A traction table (Surgical Alliance, Tokyo, Japan) [14] or a fracture table (Takara Belmont, Osaka, Japan) [15] was used for all procedures. TXA was administered before skin incision, and the femoral stem was implanted through the interval between the tensor fascia latae and the sartorius muscles, with meticulous closure of the anterior joint capsule after implant placement.

### Evaluation

Baseline characteristics and surgical data were collected, including age, sex, height, weight, body mass index (BMI), American Society of Anesthesiologists (ASA) classification, surgery duration, underlying diseases, classification of femoral stem, medical histories (autoimmune diseases, cardiovascular disease, peripheral vascular disease, deep vein thrombosis, and pulmonary embolism), intraoperative blood loss, volume of collected blood, and transfusion volume. The postoperative outcomes assessed were the inflammatory response, pain level, and hidden blood loss (HBL). The inflammatory response was evaluated by measuring serum C-reactive protein (CRP) levels on postoperative days (PODs) 1, 3, and 7. Pain levels were evaluated using a numerical rating scale (NRS) during rehabilitation sessions based on the physical therapists'chart documentation on days 1, 3, and 7. HBL was calculated as total blood loss (TBL) minus intraoperative blood loss. The TBL was estimated using Nadler’s method [16] as follows:

$$\mathrm{TBL}\;\left(\mathrm{mL}\right)=\mathrm{blood}\;\mathrm{volume}\;\left(\mathrm{BV},\mathrm L\right)\;\times\;1000\;\times\;\left(\mathrm{preoperative}\;\mathrm{Hb}-\mathrm{postoperative}\;\mathrm{Hb}\right)/\mathrm{preoperative}\;\mathrm{Hb}\;+\;\mathrm{transfusion}\;\mathrm{volume}\left(\mathrm{mL}\right)$$

where BV (L) was estimated using the formula:

$$\mathrm{BV}\left(\mathrm L\right)={\mathrm K}_1\;\times\;\mathrm{height}\left(\mathrm m\right)^2\;+\;{\mathrm K}_2\times\;\mathrm{weight}\;\left(\mathrm{kg}\right)+{\mathrm K}_3$$

where K₁ = 0.3699, K₂ = 0.03219, and K₃ = 0.6041 for men, and K₁ = 0.3561, K₂ = 0.003308, and K₃ = 0.1833 for women.

If HBL was negative, it was considered as 0.

Patients were categorized into two groups according to the use of TXA. Group A received TXA, and Group B did not receive TXA.

The primary endpoints were postoperative CRP and NRS as compared between groups A and B.

The secondary endpoints included postoperative HBL and postoperative complications, as compared between groups A and B.

### Statistical analysis

Data are presented as the mean ± standard deviation (SD) or median values. The normality of data distribution was assessed using the Shapiro–Wilk test, and variance homogeneity was evaluated using Levene’s test. Intergroup comparisons of normally distributed data were performed using the independent Student’s *t*-test, whereas binary data were analyzed using the chi-square test. Non-normally distributed data were compared using the Mann–Whitney U test.

To address potential temporal bias arising from the non-randomized design, surgery month was included as a continuous covariate in all regression models.

Postoperative outcomes were assessed in a blinded manner. NRS scores were recorded by physical therapists who were unaware of the treatment allocation. CRP levels were measured using an automated laboratory analyzer, with no investigator involvement in this process, to ensure that outcome assessment remained blinded.

Although multiple comparisons were performed across several time points, no formal correction for multiple testing was applied because the primary outcomes (postoperative CRP levels and NRS scores) were pre-specified. The results, especially those with borderline *p*-values, were interpreted with appropriate caution.

Data were analyzed using IBM SPSS Statistics (version 29.0; IBM, Armonk, NY, USA). Statistical significance was set at *P* < 0.05. Missing data were handled using complete case analysis.

Sample size estimation was performed using G*Power version 3.1.9.7. Based on prior studies reporting significant reductions in CRP levels with TXA administration in THA [17, 18], a medium effect size (Cohen’s d = 0.5) was assumed.

Using a one-tailed independent samples *t*-test with α = 0.05 and power = 0.80, the estimated sample size was 51 per group (*n* = 102). The final sample size in this study was 126 patients (TXA group = 60, control group = 66), resulting in a post hoc power of 0.87.

## Results

### Patient characteristics and surgical data

The baseline patient characteristics and surgical data are summarized in Table 1. No significant differences were observed among the groups in terms of age, sex, BMI, surgical side, ASA, underlying diseases, and medical histories (autoimmune diseases, cardiovascular disease, peripheral vascular disease, deep vein thrombosis, and pulmonary embolism). Surgical duration, intraoperative blood loss, and classification of femoral stems showed no significant differences. All patients received cementless cups, with fully porous-coated stems used in 89 cases (70.6%), single-wedge stems used in 8 cases (6.3%), partial collum short-stems used in 12 cases (9.5%), and cement stems used 16 cases (12.7%) and rectangular stem used 1 case (0.01%).

**Table 1:** Baseline characteristics and intraoperative demographics

Variables	Group A (*n* = 60)	Group B (*n* = 66)	*P*-value*
Age (SD)	68.12 (8.59)	67.44 (9.36)	0.336
Sex (male/female) (no.)	12/48	16/50	0.56
BMI (kg/m^2^) (SD)	24.23 (4.32)	23.72 (3.36)	0.23
Operated side (left/right) (no.)	28 (32)	38 (28)	0.22
ASA classification, *n* (%)			0.248
I	9 (15)	7 (16.7)	
II	50 (83.3)	54 (81.8)	
III	1 (1.7)	5 (7.6)	
Medical history			
autoimmune diseases	11	7	0.218
cardiovascular disease	3	5	0.514
peripheral vascular disease	2	0	0.176
deep vein thrombosis	0	2	0.176
pulmonary embolism	0	0	1
Classification of the femoral stem, *n* (%)			0.13
fully porous-coated stem	48 (80)	41 (62.1)	
single wedge stem	3 (5)	5 (7.6)	
partial collum short stem	4 (6.7)	8 (12.1)	
cement stem	4 (6.7)	12 (18.2)	
rectangular stem	1 (1.7)	0 (0)	
Underlying diseases, *n* (%)			0.069
osteoarthrosis	57 (95)	53 (80.3)	
femoral head necrosis	2 (3.3)	11 (16.7)	
rapid destructive coxarthrosis	1 (1.7)	1 (1.5)	
femoral neck fracture	0 (0)	1 (1.5)	
Surgical time (min) (SD)	95.51 (20.2)	89.75 (21.89)	0.065
Surgical blood loss (ml) (SD)	272.32 (93.01)	284.89 (94.98)	0.455

^*^The unpaired *t*-test was used for the analysis of age, BMI, surgical time, and surgical blood loss. The Mann–Whitney U test for sex, operated side, and medical history, and the chi-squared test for ASA classification, Classification of the femoral stem, and underlying diseases

### Postoperative inflammatory response

**Fig. 2:**
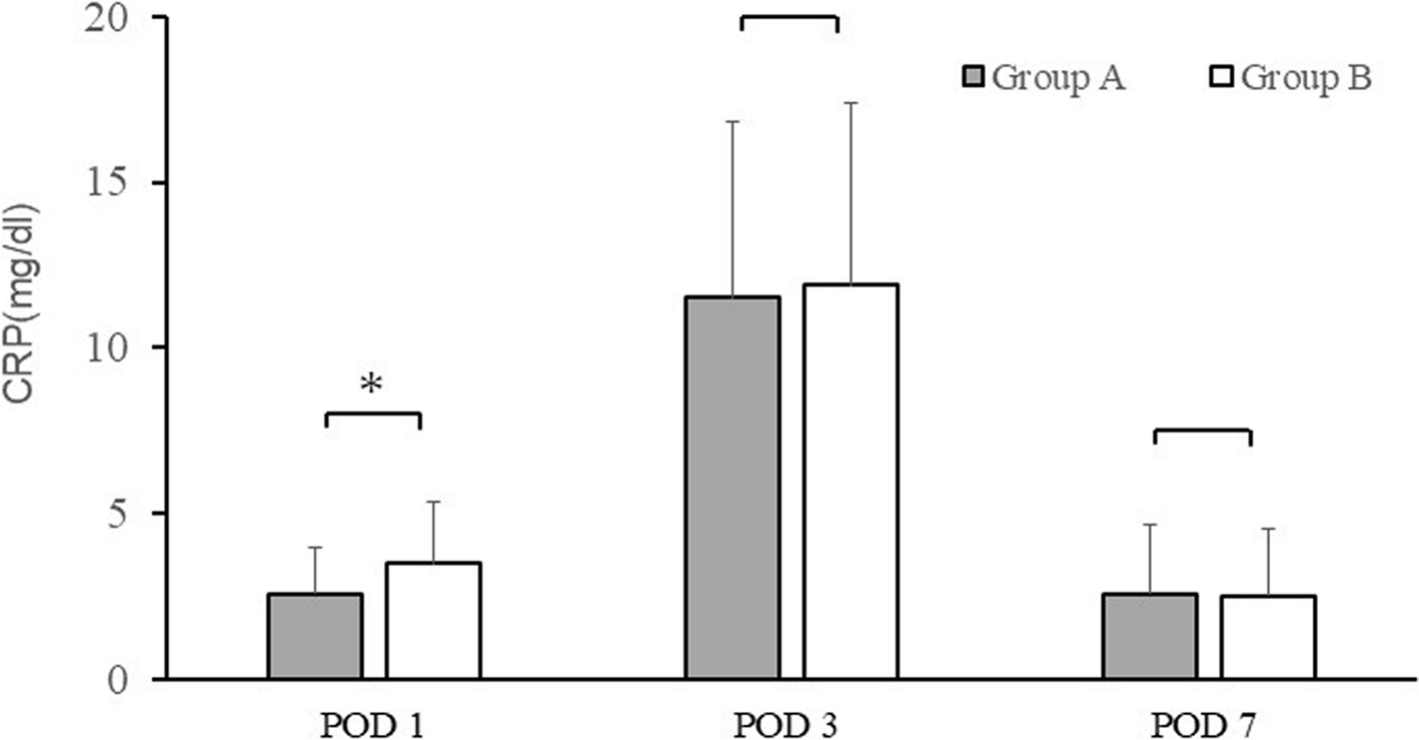
Graphs showing the postoperative serum levels of C-reactive protein. Comparison of Group A (TXA) and Group B (without TXA). The asterisks indicate values that were significantly different between groups; error bars represent the range. Post: postoperative; THA: total hip arthroplasty, TXA: tranexamic acid, CRP: C-reactive protein

The CRP trends on POD 1, 3, and 7 are shown in Fig. 2. In all groups, the CRP levels peaked on POD 3. On POD 1, the CRP levels in Group A were significantly lower than those in Group B (Group A: 2.59 ± 1.39 mg/dL, Group B: 3.48 ± 1.91 mg/dL; *P* = 0.002). On POD 3 and 7, there was no statistically significant difference between Group A and Group B (POD3: Group A: 11.52 ± 5.34 mg/dL, Group B: 11.88 ± 5.49 mg/dL; *P* = 0.354; POD 7: Group A: 2.58 ± 2.09 mg/dL, Group B: 2.52 ± 2.02 mg/dL; *P* = 0.428).

The month of surgery was not significantly associated with CRP on PODs 1, 3, 7 (*P* = 0.23, 0.071, 0.203), suggesting that the results were not affected by temporal bias.

### Postoperative pain

**Fig. 3:**
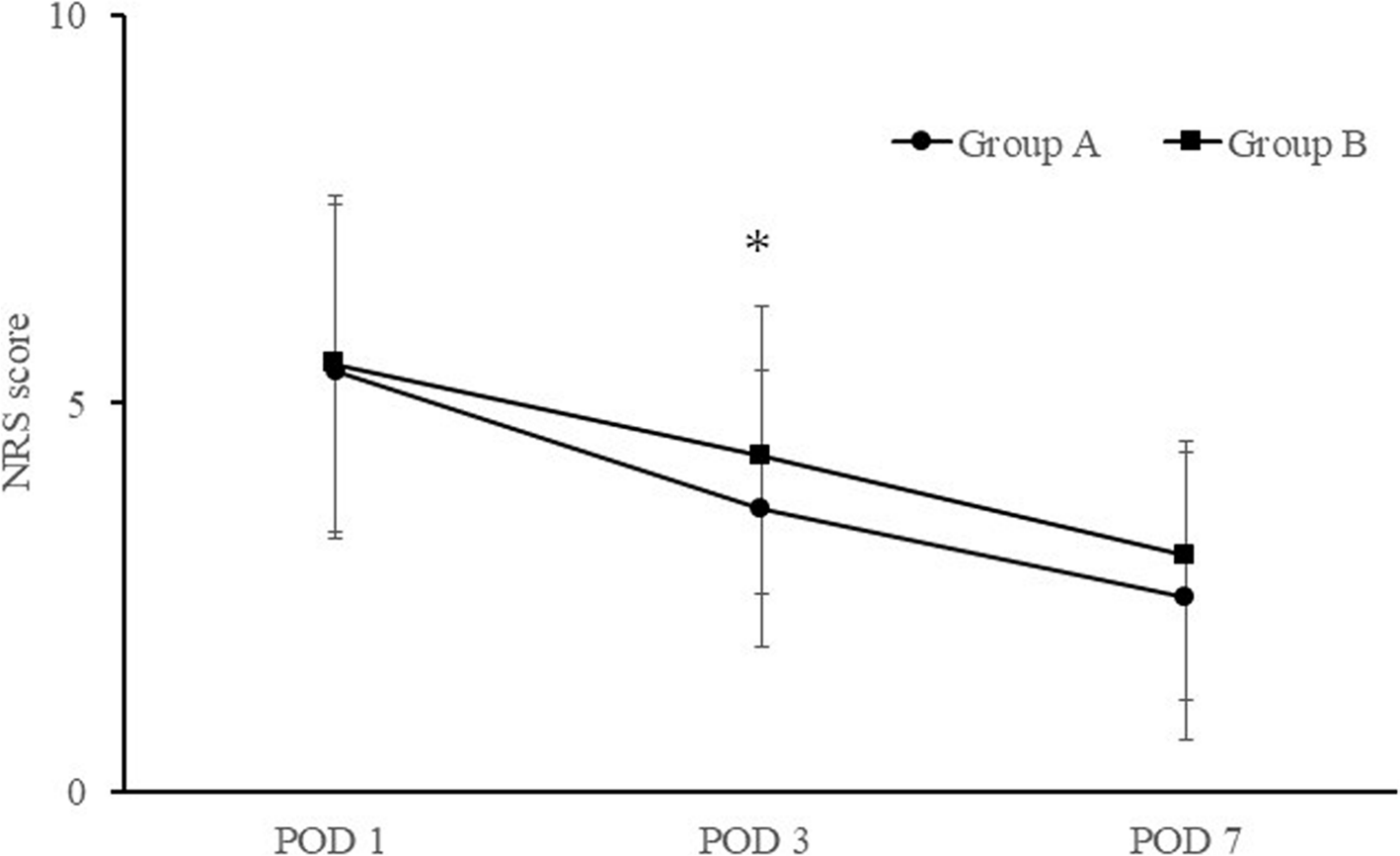
Graphs showing the postoperative Numerical Rating Scale scores. Comparison of Group A (TXA) and Group B (without TXA). The asterisks indicate values that were significantly different between groups; error bars represent the range. Post: postoperative; THA: total hip arthroplasty, TXA: tranexamic acid, NRS: Numerical Rating Scale

NRS score trends on PODs 1, 3, and 7 are summarized in Fig. 3. In all groups, the NRS levels peaked on POD 1. On POD 3, NRS scores were significantly lower in Group A than in Group B (Group A: 3.65 ± 1.92, Group B: 4.32 ± 1.78; *P* = 0.031). Although NRS scores tended to be lower in Group A than in Group B on PODs 7, the differences were not statistically significant (Group A: 2.52 ± 1.46, Group B: 3.04 ± 1.86, *P* = 0.065). On POD 1, there was no statistically significant difference between Group A and Group B (Group A: 5.4 ± 2.18, Group B: 5.5 ± 2.15, *P* = 0.407).

The month of surgery was not significantly associated with NRS scores on PODs 1, 3, 7 (*P* = 0.743, 0.233, 0.695), suggesting that the results were not affected by temporal bias.

### Postoperative HBL

**Fig. 4:**
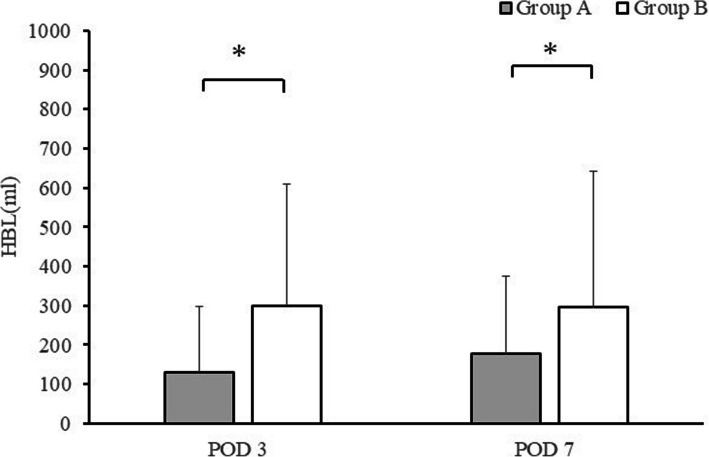
Graphs showing the postoperative HBL. Comparison of Group A (TXA) and Group B (without TXA). The asterisks indicate values that were significantly different between groups; error bars represent the range. Post: postoperative; THA: total hip arthroplasty, TXA: tranexamic acid, HBL: hidden blood loss

The HBL trends on PODs 3 and 7 are shown in Fig. 4. In all groups, HBL levels were higher on POD 7 than on day 3. On PODs 3 and 7, HBL levels in Group A were significantly lower than those in Group B (POD 3: Group A: 130.8 ± 166.49 mL, Group B: 300.32 ± 308.32 mL; *P* = 0.001; POD 7: Group A: 176.89 ± 197.26 mL, Group B: 295.1 ± 347.19 mL; *P* = 0.013).

The month of surgery was not significantly associated with HBL on postoperative 3, 7 (*P* = 0.57, 0.69), suggesting that the results were not affected by temporal bias.

### Complications

No deep vein thrombosis, pulmonary embolism, or wound complications were observed in any group. However, one case of urinary tract infection during hospitalization was observed in Group A.

## Discussion

The effects of TXA alone on inflammation, postoperative pain, and HBL in THA were investigated. We found that CRP levels were significantly lower in the group receiving TXA than in the group that did not receive TXA on POD1. The NRS pain score on POD 3 was also lower in the group that received TXA only than in the group that did not receive TXA. HBL was lower on PODs 3 and 7 in the former group than in the latter group.

Acute postoperative pain lasting between 6 and 24 h, driven by surgical trauma [19] and the migration of inflammatory mediators [20], remains a persistent challenge. Mitigating these issues could expedite rehabilitation and enhance patient outcomes. Glucocorticoids, such as DEX, have potent anti-inflammatory properties and efficacy in managing postoperative pain, but their application is often limited by their potential side effects, including fluctuations in blood glucose levels. TXA, a synthetic lysine analog, inhibits lysine-binding sites on plasminogen, thereby reducing its conversion to plasmin and preventing fibrin degradation [21, 22]. This mechanism stabilizes fibrin clots and reduces bleeding. With a half-life of approximately 2 h, TXA exhibits antifibrinolytic effects in the bloodstream for 7–8 h and in tissues for up to 17 h [23]. Additionally, TXA has demonstrated anti-inflammatory properties, which are exerted by suppressing the plasmin-mediated activation of complement pathways, monocytes, and neutrophils [9]. Although the combined effects of TXA and DEX in terms of anti-inflammatory benefits in THA have been explored [18], this study is the first to evaluate the anti-inflammatory effects of TXA monotherapy in DAA-THA. Given that DAA is a minimally invasive technique involving an intermuscular plane, the perioperative inflammatory response may differ from that observed with other surgical approaches. Our findings offer valuable insight into inflammation management strategies in the context of minimally invasive hip arthroplasty.

Furthermore, additional maintenance doses following preoperative administration may enhance the anti-inflammatory effects of TXA [24]. However, TXA doses exceeding 3000 mg are associated with an elevated risk of seizures [25]. In our study, a single preoperative dose of 1000 mg was administered. This approach significantly reduced CRP levels on POD 1 compared with the group that did not receive TXA. Interestingly, pain relief was more evident on POD 3 than on POD 1. This temporal discrepancy may be due to the delayed peak of postoperative pain, which is often influenced by factors such as HBL, local swelling, and soft tissue tension. These factors tend to peak around PODs 2 to 3 [26], contributing to increased pain perception during this period.

DEX, a long-acting glucocorticoid with potent anti-inflammatory properties, is effective in reducing postoperative pain, nausea, and vomiting. The intraoperative use of DEX in THA does not increase the risk of gastrointestinal bleeding or wound complications [27, 28]. Furthermore, studies on the combined use of TXA and DEX have demonstrated favorable outcomes [11]. Nevertheless, the potential risks associated with DEX, such as immunosuppression, warrant careful consideration, particularly in patients with a risk of infection. In this study, the effects of TXA alone on inflammation, postoperative pain, and HBL in THA were indicated.

Although several studies have reported that the combination of TXA and glucocorticoids results in superior outcomes compared to TXA alone—such as significantly lower CRP and interleukin-6 (IL-6) levels and improved postoperative pain scores [29]—the use of glucocorticoids may not be suitable for all patients due to the risk of immunosuppression or other contraindications.

If there is hesitation in using glucocorticoids during THA, the use of TXA alone may be a viable option.

While previous studies have mainly focused on the combined use of TXA with corticosteroids, these results suggest that TXA alone can offer substantial benefits in reducing inflammation, pain, and blood loss following DAA-THA. These findings support the clinical viability of TXA monotherapy, especially within the context of minimally invasive hip arthroplasty.

### Study Limitations

This study had some limitations. The retrospective nature of the study did not allow the establishment of causal relationships. Moreover, the follow-up period was confined to the hospitalization phase, precluding the assessment of medium to long-term safety outcomes. No TXA-related adverse events were observed, and the safety profile of TXA is well supported by meta-analyses of various dosing regimens and administration routes [30, 31]. Different stem types were employed in THA. However, no significant differences were observed between Groups A and B, minimizing the potential impact on the study outcomes.

Although statistically significant differences were observed in certain postoperative outcomes, including NRS on POD 3, CRP on POD 1, and HBL, these analyses were exploratory and involved multiple comparisons without formal adjustment. Therefore, the findings should be interpreted with caution and regarded as hypothesis-generating rather than confirmatory. Further studies with larger sample sizes and predefined primary outcomes are warranted to validate these results.

## Conclusions

In this study, it was found that the use of single-dose TXA (1000 mg IV) in DAA-THA significantly reduced postoperative inflammation, pain, and HBL. These findings suggest that TXA administration may facilitate early rehabilitation following surgery. We recommend the routine use of single-dose TXA (1000 mg IV) in DAA-THA to optimize early postoperative recovery.

## Data Availability

No datasets were generated or analysed during the current study.

## Abbreviations

BMI: Body mass index
CRP: C-reactive protein
DEX: Dexamethasone
HBL: Hidden blood loss
THA: Total hip arthroplasty
TXA: Tranexamic acid
POD: Postoperative day
DAA: Direct anterior approach
